# Vaginal aging: from concealed symptoms to defined biomarkers

**DOI:** 10.1038/s41514-026-00404-7

**Published:** 2026-05-15

**Authors:** Tong Wu, Yibao Huang, Rui Li, Yin Xie, Mingfu Wu

**Affiliations:** 1 Key Laboratory of Cancer Invasion and Metastasis, Ministry of Education, Tongji Hospital, Tongji Medical College, Huazhong University of Science and Technology, Wuhan, China; 2 Department of Obstetrics and Gynecology, Tongji Hospital, Tongji Medical College, Huazhong University of Science and Technology, Wuhan, China; 3 National Clinical Research Center for Obstetrical and Gynecological Diseases, Tongji Hospital, Tongji Medical College, Huazhong University of Science and Technology, Wuhan, China; 4 Emergency Department, Tongji Hospital, Tongji Medical College, Huazhong University of Science and Technology, Wuhan, China; 5 Department of Obstetrics, Maternity and Child Health Care Hospital of Hubei Province, Wuhan, China

**Keywords:** Biomarkers, Diseases, Health care, Medical research

## Abstract

Vaginal aging is a multifactorial biological process characterized by structural, functional, and molecular alterations driven primarily by estrogen decline. This review summarizes current evidence on potential biomarkers across five domains: physiological parameters, imaging features, histological changes, and molecular alterations. We further discuss methodological challenges and future research directions necessary to establish standardized and clinically applicable biomarkers. Understanding these biological indicators may facilitate earlier diagnosis, improve patient stratification, and enable personalized therapeutic interventions in women experiencing vaginal aging.

## Introduction

The vagina is an elastic muscular tube that extends from the vulva to the cervix of the uterus. Its unique structural composition includes fibrous adventitia, smooth muscle layer, and mucosal lining with transverse folds, allowing it to accommodate various physiological changes, sexual intercourse, childbirth, and maintain pelvic organ support^1^. This adaptability underscores the vagina’s essential role throughout a woman’s life, especially concerning reproductive health and sexual function.

**Fig. 1:**
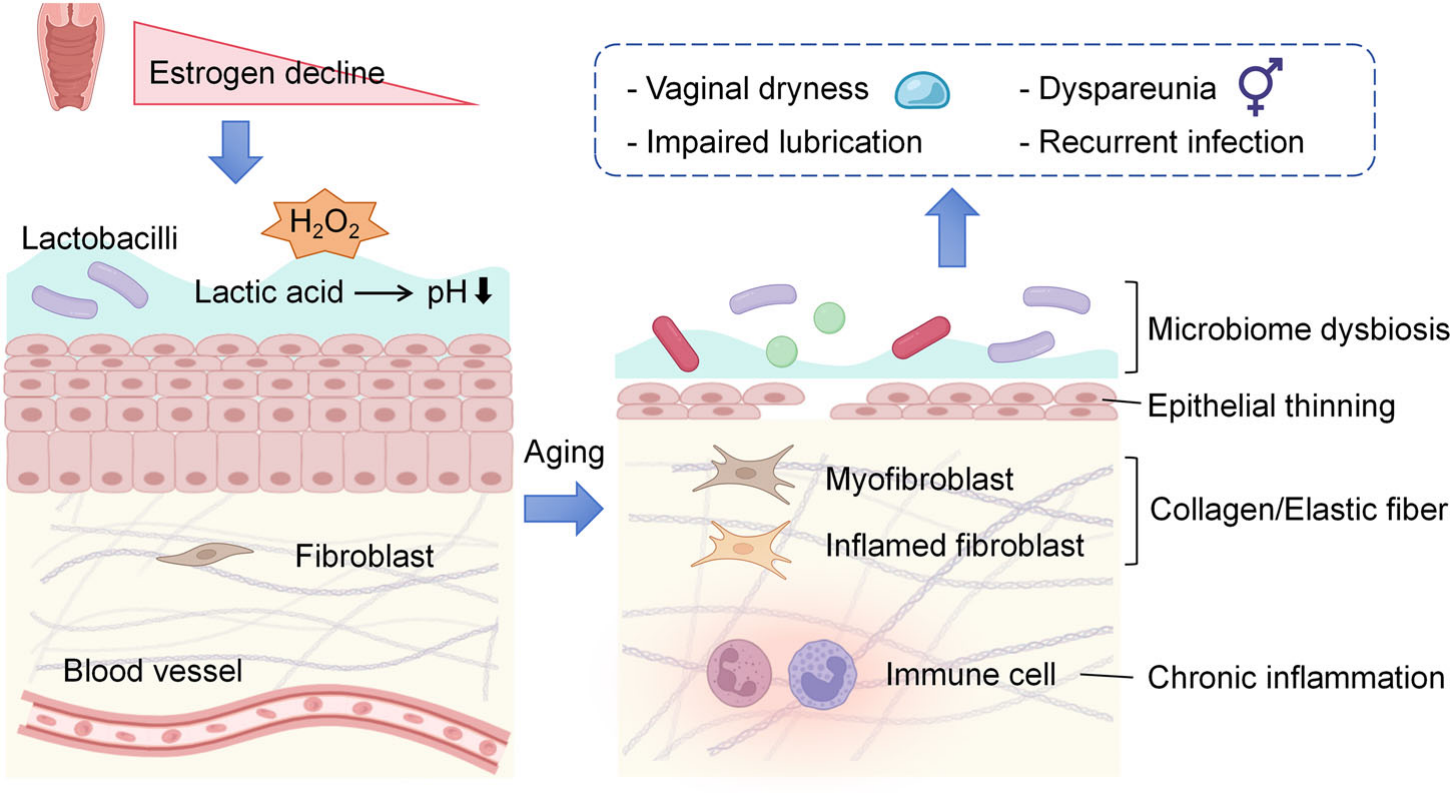
Schematic overview of vaginal aging mechanisms. Estrogen decline drives progressive structural and functional deterioration of the vaginal wall. Young vaginal tissue is characterized by a Lactobacillus-dominant microbiome, thick epithelium, and intact vascularity. Aging induces microbiome dysbiosis, epithelial thinning, fibroblast activation, extracellular matrix remodeling, and chronic inflammation, ultimately leading to clinical symptoms.

Vaginal aging is a multifactorial biological process characterized by structural, functional, and molecular alterations driven primarily by estrogen decline^2^. Chronic inflammation, oxidative stress, genetic predisposition, and adverse lifestyle factors may also accelerate vaginal aging^3,4^. Vaginal aging usually causes vulvovaginal dryness, dyspareunia, and recurrent infections, as well as long-term impacts on sexual quality and psychological well-being (Fig. 1)^5–7^. However, research into biomarkers for characterizing this process remains notably underdeveloped, representing a critical barrier to clinical progress. Identifying reliable, specific, and clinically applicable vaginal aging biomarkers is crucial for early detection, accurate assessment, and effective intervention of age-related vaginal disorders^8^.

Vaginal aging biomarkers are measurable biological indicators reflecting structural, functional, or molecular alterations associated with the aging of vaginal tissues. They can: (i) reflect age-related alterations in the vagina, (ii) track the course of vaginal aging, and (iii) forecast the transition from physiological to pathological states^9^. Specifically, these biomarkers should identify vaginal-unique changes^10^, and discriminate between physiological aging and pathological conditions^11^. Considering that the vagina also interacts with the uterus, ovaries, and urinary bladder via hormonal^12,13^ and microbiota approaches^14^, the biomarkers should also mirror the potential systemic impacts on the vagina. Additionally, vaginal biomarkers ought to be detectable through non-invasive or minimally invasive approaches to enable longitudinal monitoring and clinical use.

In this review, we clarify the significance of vaginal aging and its involved population. We summarize the current evidence on vaginal aging biomarkers, encompassing five core pillars: physiological characteristics, medical imaging traits, histological features, and molecular alterations. Additionally, we critically discuss unresolved challenges and the need to explore systemic links between vaginal aging and other age-related diseases. Our goal is to provide a comprehensive reference for researchers, clinicians, and public health professionals working in the women’s health and aging domain.

## Literature search strategy

Relevant studies were identified through searches in PubMed, Web of Science, Scopus, and Embase databases up to October 2025. A combination of domain-specific keywords was used for retrieval. For vaginal aging, the search terms included “vaginal aging”, “vaginal atrophy”, “vulvovaginal atrophy”, “genitourinary syndrome of menopause”, and “GSM”. For biomarkers, the keywords covered “biomarker”, “biological marker”, “omics”, “transcriptomics”, “proteomics”, “metabolomics” and “microbiome”. All authors participated in the entire literature search process to ensure the objectivity and accuracy of retrieval. Only English-language articles that reported physiological, imaging, histological, or molecular features of vaginal aging were considered.

After the initial literature screening based on titles and abstracts, full-text evaluations of potential eligible studies were conducted by two independent authors (T.W. and Y.X.). The following key information were extracted: author, publication year, research type, sample size, grouping, main findings, and methodological limitations (Table 1, Table 2). Any discrepancies were resolved through discussion with a third senior author (M.F.W.) to reach a consensus.

**Table 1:** Evidence and critical appraisal for vaginal aging biomarkers

Author	Research type	Sample size	Main findings	Method limitation
**Clinical Symptoms: Vaginal Dryness and Impaired Lubrication**				
A.B. Handy (2020)	Clinical study	Women with sexual arousal concerns (n = 32) and without (n = 32)	Schirmer Tear Test strips detect significant increases in vaginal lubrication post-sexual film exposure (pre-film mean 3.19 mm vs. post-film 6.27 mm).	Self-administered test strips may have insertion variability affecting accuracy.
D. Gabrieli (2022)	Clinical study	GSM group (n = 30), control group (n = 28)	Modified Schirmer test distinguishes GSM-related vaginal dryness: control group mean measurement (21.7 mm) vs. GSM group (3.3 mm).	Lack of pre/post-treatment measurements to validate utility in treatment evaluation.
**Clinical Symptoms: Dyspareunia**				
D.A. Revicki (2011)	Clinical study	Premenopausal women with HSDD (n = 15), Premenopausal women with HSDD (n = 30), postmenopausal women with HSDD (n = 31)	80–100% of women found all FSFI items clear and easy to understand; 93–100% rated the FSFI desire domain items as clear and relevant.	Content validity not examined in women with normal sexual function.
A.C. Daescu (2023)	Clinical study	FSD group (n = 158), control group (n = 227)	FSFI-RO (Romanian version) has high internal consistency (Cronbach’s α = 0.944) and test-retest reliability (ICC = 0.942–0.991).	Constructs like sexual well-being and self-esteem not measured to enhance construct validity.
M. Gabes (2021)	Clinical study	Postmenopausal women with GSM-related vaginal symptoms (n = 185)	Refined German DIVA questionnaire has 17-item short-version and 21-item long-version.	No clinical/laboratory confirmation of GSM.
A.J. Huang (2015)	Clinical study	Symptomatic postmenopausal women (n = 745)	DIVA questionnaire has 4 domains with good model fit (CFI = 0.975–0.987, SRMR = 0.038–0.048).	Cross-sectional design prevents assessment of symptom impact over time.
M.M. Hunter (2016)	Clinical study	Symptomatic postmenopausal women (sexually active n = 462, non-sexually active n = 283)	Depression and weekly urinary incontinence are strong predictors of greater vaginal symptom impact.	Overall DIVA scores are low (mean <1.0/4), limiting generalizability to women with severe vaginal symptoms.
B. Sert (2022)	Clinical study	Postmenopausal women with vaginal symptoms (n = 218)	The Turkish DIVA has excellent reliability: Cronbach’s α = 0.87–0.96, item-total correlations=0.60–0.91, and test-retest ICC = 0.90–0.99.	No objective assessments to confirm symptom etiology or pelvic floor status.
P.I. Hadiprajitno (2024)	Clinical studies (scale development)	Sexually active Indonesian women	ISQOL-W has strong psychometrics: Cronbach’s α = 0.915 (overall), 0.826-0.903 (subscales).	No confirmatory factor analysis performed; larger diverse sample needed for validation.
M.M. Hunter (2020)	Clinical study	Postmenopausal women with vulvovaginal symptoms (n = 301)	DIVA domains show strong internal consistency (Cronbach’s α = 0.82-0.90) and item-scale correlations >0.60.	No data on minimal clinically important differences for DIVA scores.
M. Gabes (2021)	Clinical study	Postmenopausal women with GSM symptoms (online n = 144, paper-pencil n = 41)	German DIVA confirms 4-factor structure with strong internal consistency (Cronbach’s α = 0.84-0.93).	No sexual function reference measure to validate DIVA sexual functioning domain.
**Biochemical biomarkers: Vaginal pH**				
J. Mania-Pramanik (2008)	Clinical study	control group n = 49, LGTIs n = 185, RSA n = 45, infertility n = 75	Vaginal pH >4.5 is strongly correlated with bacterial vaginosis with 100% positive predictive value and 92.5% negative predictive value.	Estrogen levels were not measured, which may influence vaginal pH.
S. Bakir (2021)	Clinical study	n = 97 (46.4% menopausal, 14.4% on hormone replacement therapy)	Adding sterile saline to vaginal discharge artificially increases pH.	No assessment of inter-observer reliability for pH readings.
H. Li (2021)	Laboratory studies (technical development)	Standard solutions: pH 1.40–2.35 (20 samples), 2.90–5.20 (28 samples), 7.90–9.70 (19 samples); Real-world samples: vinegar, lemonade, soda (3 samples)	Developed “Smart-pH-Reader” (smartphone + 3D-printed optical accessory) using dominant wavelength analysis of commercial test strips.	Performance degrades with colored samples due to interference with colorimetric analysis.
X.L. Xiong (2024)	Laboratory studies (technical development)	Standard solutions: n = 210 (pH 5.0–6.0, 7.0–8.0, 0.05 interval); Real-world samples: 4 (3 PBS buffers, 1 river water)	Developed machine learning model combining SHAP-based feature selection and Stacking fusion.	Limited to pH range 5.0–8.0; untested for extreme acidic/alkaline conditions.
**Biochemical biomarkers: Metabolites**				
X. Zhang (2025)	Clinical study	Reproductive-aged (n = 5), postmenopausal (n = 5); multi-omics analysis: n = 5 per group for each omics layer	Multi-omics integration identifies 3 key aging-related pathways: glycolysis, estrogen signaling, and oxidative stress	Lack of functional validation for key pathways.
H.M. Jones (2022)	Cellular experiment; clinical sample	Premenopausal (n = 15), postmenopausal (n = 15)	Postmenopausal vaginal fluid has lower L-lactate and D-lactate than premenopausal samples.	No assessment of lactate’s effect on vaginal microbiome.
**Imaging Biomarkers: Traditional Ultrasound**				
S. Türk (2022)	Clinical study	Postmenopausal group (n = 80), premenopausal group (n = 80)	Total vaginal wall thickness and total mucosal thickness in postmenopausal women are significantly lower than those in premenopausal women.	Potential effects of menstrual cycle phase, BMI, and other factors on vaginal thickness were not analyzed.
A. Balica (2017)	Clinical study	Premenopausal group (n = 54), postmenopausal group (n = 21)	Total vaginal wall thickness in premenopausal women (15.3 mm) is significantly higher than that in postmenopausal women (12.3 mm).	Interobserver repeatability and measurement stability were not assessed.
G.M.V. Pereira (2022)	Clinical study	Women with vaginal laxity (n = 162)	Significant differences in vaginal wall thickness measurements between transabdominal ultrasound and transvaginal ultrasound were found in the proximal, middle-third, and distal compartments.	Interobserver reliability was not assessed.
H. Peker (2021)	Clinical study	GSM group (n = 20), non-GSM group (n = 20)	3D EVUS can be a useful non-invasive tool for the objective diagnosis of GSM.	Effects of ethnicity and anatomical differences on vaginal wall thickness were not considered.
J.L. Alcazar (2023)	Clinical study	Premenopausal women (n = 10)	A standardized method for measuring vaginal wall thickness using 3D transvaginal ultrasound is proposed.	3D ultrasound equipment and specialized software are not available in all laboratories, limiting promotion.
M.A. Weber (2016)	Clinical study	VA group (n = 8), control group (n = 9)	Vaginal focal depth measurement is a new noninvasive method to quantify vaginal wall thickness.	Lack of intra- and interobserver variability analysis, and no comparison with other vaginal wall thickness measurement methods.
**Imaging Biomarkers: Elasticity Imaging Technology**				
V. Egorov (2012)	Clinical study	Normal pelvic support group (n = 18), pelvic organ prolapses (POP) group (n = 13)	Vaginal tactile imaging (VTI) enables 3D imaging of the vagina and quantitative assessment of tissue elasticity.	Patient movement during examination may produce image artifacts, affecting the accuracy of elasticity measurements.
**Histological Biomarkers: Epithelial Layer Changes**				
S. Wan (2022)	Animal model	Rptor cKO mice (n = 3-15), OVX mice (n = 3-7)	Rptor deficiency disrupts estrous cycle homeostasis, leads to vaginal atrophy via promoting cell death and inhibiting proliferation.	Cannot accurately clarify the exact role of mTOR in stromal cells and epithelia separately.
M.M. Kajikawa (2014)	Clinical study	Neovagina group (n = 6), control group (n = 10)	ERα expression in neovaginal tissue is significantly lower than in control premenopausal women	Small sample size (only 6 valid cases in neovagina group).
A. Hofsjö (2017)	Clinical study	Cervical cancer survivors (n = 34), age-matched controls (n = 37)	Survivors have shorter vagina (7.0 cm vs. 10.3 cm in controls), 91% vaginal atrophy, and 97% pelvic fibrosis.	Most survivors underwent surgery/chemotherapy in addition to radiotherapy, confounding effects.
M.d.S.D. Babinski (2023)	Clinical study	Pre-menopausal (pre-M, n = 10), post-menopausal (post-M, n = 10)	Post-M samples exhibit collagen fibril fusion, plate formation, and disorganized 3D structure in the subepithelium.	No quantitative analysis of collagen subtypes or ultrastructural details.
Z. Xiao (2025)	Clinical study	10 per age group: 20–30 years, 30–40 years, 40–50 years, >50 years	Epithelial thickness decreases linearly with age (586.5 → 309.5 μm, 47.3% reduction, p < 0.0001).	No analysis of collagen ultrastructure or molecular mechanisms.
M. Baldassarre (2015)	Clinical study	Post-menopausal women: No diabetes (n = 11), Type 2 diabetes (n = 10)	Diabetic women have increased but morphologically disrupted microvessel density in vaginal lamina propria.	Limited to post-menopausal women; results may not apply to pre-menopausal diabetics.
**Histological Biomarkers: Muscular Layer Changes**				
S.E. White (2024)	Animal model	Nulliparous CD-1 mice (n = 64)	Vaginal contractility decreases with age: Circumferential and longitudinal contractile potential decline progressively.	Limited to nulliparous mice.
**Molecular Biomarkers: Epithelial Cells**				
J. Zhu (2015)	Animal model	Control (n = 6), ovariectomy (n = 6), ovariectomy+estrogen therapy (n = 6)	Serum estrogen level is positively correlated with AQP expression and vaginal lubrication.	Lack of direct evidence for causal link between AQP reduction and decreased lubrication.
Y. Zhao (2024)	Cellular experiment	VK2/E6E7 vaginal epithelial cells; control, C. albicans-infected, and amphotericin B-treated groups (n = 6 per group)	Infection inhibits LDHA and PKM2 expression, decreases glycolytic metabolites.	No assessment of clinical relevance of identified metabolites.
A. Hodonua (2019)	Animal models; cellular experiment	Animal: Mink (n = 12); Cellular: GMMe mink uterine epithelial cells (n = 3 per treatment group)	Insulin up-regulates Insr mRNA 2-fold; P₄ reduces Insr expression to control levels.	No assessment of glycogen metabolism in vaginal vs. uterine epithelium.
D. Li (2023)	Animal models; cellular experiment	Animal: Sprague-Dawley rats (n = 24); Cellular: VK2/E6E7 cells (n = 6 per treatment group)	Vitamin D supplementation reduces vaginal pH, increases uterine/vaginal weight, promotes epithelial proliferation and keratinization.	No direct comparison of vitamin D vs. E₂ efficacy.
A. Lee (2017)	Clinical studies; cellular experiment	Clinical: Premenopausal (PRE, n = 19), postmenopausal atrophic vagina (PAV, n = 13), postmenopausal non-atrophic (PON, n = 4) women; Cellular: VK2/E6E7 cells (n = 3 per treatment group)	p-RhoA and p-Ezrin expression is higher in PRE and PAV vs. PON vaginal tissue.	No assessment of PAV symptom severity correlation with p-RhoA/p-Ezrin levels.
**Molecular Biomarkers: Fibroblasts**				
O. Shynlova (2013)	Clinical study	Premenopausal secretory (n = 10), premenopausal proliferative (n = 8), postmenopausal control (n = 5), postmenopausal POP (n = 13)	Menstrual cycle phase modulates ECM genes: MMP1 downregulated, TIMP1/LOXL4 upregulated in proliferative vs. secretory phase.	No protein-level validation of gene expression data.
Y. Wen (2013)	Cellular experiment	Vaginal fibroblasts from 2 women: 78-year-old (POP + SUI) and 47-year-old (non-POP)	Reprogramming efficiency higher in younger donor (76% fully reprogrammed colonies) vs. older donor (56%).	No functional assessment of redifferentiated fibroblasts in tissue repair models.
A.M. Ruiz-Zapata (2020)	Cellular experiment	Vaginal tissues from POP and healthy women; Cellular: vaginal fibroblasts (POP/healthy) seeded on stiffness gradients	Matrix stiffness positively correlates with α-SMA expression in both POP and healthy fibroblasts, but not collagen production.	In vitro model lacks physiological mechanical forces.
**Molecular Biomarkers: Immune Cells**				
E.A. Kremleva (2016)	Clinical studies; cellular experiment	healthy women (n = 24), bacterial vaginosis (n = 32)	Vaginal concentrations of IL-1β, IL-8, IL-6 are higher in dysbiosis vs. normocenosis	Limited to reproductive-aged women.
M.C. Latorre (2022)	Animal model	Ovarian cycle phases: diestrus, proestrus, estrus, metestrus (n = 5–12 per group)	Vaginal neutrophil infiltration is highest in diestrus, absent in estrus; independent of pathogen/sperm challenge.	No assessment of long-term STI susceptibility during estrus.
S.E. Millar (2021)	Clinical study	healthy premenopausal (n = 4), healthy postmenopausal (n = 4), postmenopausal with atrophy (n = 4)	Identifies 12 major vaginal cell types, with epithelial cell subclusters showing the most aging-related changes.	High cost and technical complexity of single-cell sequencing limit scalability.
**Molecular Biomarkers: Metagenomic Alterations**				
H.N. Hamza (2025)	Clinical study	premenopausal (n = 25), perimenopausal (n = 25), menopausal (n = 25), postmenopausal (n = 25)	Postmenopausal women have significantly higher diverse bacterial growth vs. premenopausal, with reduced Lactobacillus acidophilus.	Reliance on culture-based methods may miss unculturable microorganisms.
H.T. Dang (2025)	Clinical study	Total n = 47 women	Aging correlates with decreased Lactobacillus crispatus levels and increased non-Lactobacillus dominant microbiomes.	Lack of menopause status documentation.
X. Li (2024)	Clinical study	childbearing (n = 6), postmenopausal (n = 6), postpartum (n = 6)	Lactobacillus dominance disappears in postpartum and postmenopausal women, with increased microbial diversity.	No assessment of clinical symptoms linked to microbiome shifts.
J.M. Bohbot (2018)	Clinical study	Total n = 98 (full analysis set, FAS);	Vaginal administration of L. crispatus IP174178 significantly reduces BV recurrence rate.	Failed to meet recruitment target, reducing statistical power for secondary endpoints.
Y. Li (2023)	Clinical study	Vaginal tissue samples: young group (n = 3), elderly group (n = 3)	Aging reduces epithelial cell subsets by 35% and increases stromal cell proportion by 28% in vaginal tissue.	Single time-point analysis; no longitudinal data on cell trajectory during aging.
J. Wang (2024)	Clinical study	aginal swabs for 16S rRNA sequencing (n = 60) and metabolomics (n = 58)	Postmenopausal GSM group has lower Lactobacillus abundance and higher Gardnerella.	Lack of external validation cohort to confirm metabolomic-microbiomic associations.

*AQP* aquaporin, *BV* bacterial vaginosis, *CFI* Comparative Fit Index, *DIVA* day-to-day impact of vaginal aging, *ECM* extracellular matrix, *FAS* full analysis set, *FSD* female sexual dysfunction, *FSFI* Female Sexual Function Index, *GSM* genitourinary syndrome of menopause, *HSDD* hypoactive sexual desire disorder, *ICC* intraclass correlation coefficient, *LGTI* lower genital tract infection, *OVX* ovariectomized, *PAV* postmenopausal atrophic vagina, *POP* pelvic organ prolapse, *PON* postmenopausal non-atrophic, *PRE* premenopausal, *RSA* recurrent spontaneous abortion, *SHAP* SHapley Additive exPlanations, *SRMR* standardized root mean square residual, *SUI* stress urinary incontinence, *VTI* vaginal tactile imaging.

**Table 2:** Summary Table of Vaginal Aging Biomarkers

Biomarker	Evidence	Clinical relevance
**Physiological type**		
Modified Schirmer test value	Clinical	Quantification of vaginal dryness severity, efficacy evaluation of GSM.
Vaginal Health Index score	Clinical	Comprehensive assessment of vaginal health, grading of aging severity.
FSFI score	Clinical	Assessment of sexual dysfunction.
DIVA questionnaire score	Clinical	Assessment of vaginal aging’s impact on daily life, psychological state and sexual function.
**Biochemical type**		
Vaginal pH	Clinical	Diagnosis of vaginal microecological imbalance, auxiliary diagnosis of GSM.
Fatty acid	Clinical + Experimental	Prediction of genital tract inflammation, assessment of aging at the metabolic level.
Glycogen/lactic acid	Clinical + Experimental	Assessment of microecological maintenance capacity.
**Imaging type**		
Vaginal wall thickness	Clinical	Objective diagnosis of GSM, assessment of structural changes in vaginal aging.
Vaginal tissue elasticity index	Clinical	Evaluation of vaginal elasticity, screening for pelvic organ prolapse risk.
**Histological type**		
Collagen Ⅰ/Ⅲ ratio	Experimental + Clinical	Assessment of vaginal wall extracellular matrix remodeling, judgment of fibrosis degree.
Vaginal epithelial thickness	Clinical	Quantification of vaginal epithelial atrophy severity, aging grading.
Estrogen receptor α	Clinical + Experimental	Assessment of estrogen sensitivity in vaginal tissue, reference for intervention targets.
**Molecular type**		
Aquaporin	Experimental	Mechanistic research related to lubrication function.
p-RhoA/p-Ezrin	Clinical + Experimental	Regulation of vaginal epithelial proliferation, reference for efficacy of estrogen replacement therapy.
MMPs/TIMPs expression	Clinical + Experimental	Assessment of vaginal wall extracellular matrix metabolism, judgment of fibrosis risk.
Lactobacillus abundance	Clinical	Diagnosis of vaginal microecological imbalance, prognosis and intervention reference for GSM.
FOLR2⁺ macrophages	Clinical	Assessment of vaginal chronic inflammatory state.
Lipid peroxide	Experimental	Assessment of oxidative stress degree.

*DIVA* day-to-day impact of vaginal aging, *FSFI* Female Sexual Function Index, *GSM* genitourinary syndrome of menopause, *MMP* matrix metalloproteinase, *TIMP* tissue inhibitors of metalloproteinase.

## Significance of Vaginal Aging

In 2014, the International Society for the Study of Women’s Sexual Health and The Menopause Society introduced the term “genitourinary syndrome of menopause” (GSM) to collectively describe vaginal, urinary, and sexual symptoms because of menopause^6^. GSM affects an estimated 40% to 60% of postmenopausal women worldwide, with vaginal aging being a key aspect^15,16^. In a multi-center observational study with 913 participants, women with GSM experienced vaginal dryness (100%), dyspareunia (77.6%), burning (56.9%), itching (56.6%), and dysuria (36.1%)^17^. However, vaginal aging-related symptoms are common but often overlooked, and not fully treated^18^. For instance, the European REVIVE survey demonstrated that vaginal dryness to be the most prevalent symptom among 3768 postmenopausal women, yet only 10% of cases were proactively discussed by healthcare professionals, and 32% of the participants reported no history of any intervention^19^. Overall, the symptoms and management of vaginal aging remain underrecognized, and relevant research is substantially limited relative to studies on other organs.

The reasons underlying the deficient focus can be attributed to societal norms, personal embarrassment, and a lack of awareness. According to the Women’s EMPOWER survey, postmenopausal women often fail to recognize vaginal aging as a chronic condition, and are reluctant to discuss these symptoms with clinicians^20^. Similarly, the CLOSER survey and the GENJA study found that many women do not communicate their experiences of vaginal discomfort to their partners or healthcare providers^21,22^. These findings underscore the need for healthcare providers to initiate conversations about vaginal health to improve awareness and treatment options.

Beyond physical symptoms, vaginal aging also affects psychological well‑being and family dynamics. Vaginal aging-related sexual dysfunction may lead to avoidance of sexual activity among women, thereby reducing self-confidence, diminishing their self-image^23^, as well as impairing intimate relationships and family life^24,25^. Additionally, gynecological cancer survivors, who experience pathological vaginal aging resulting from the disease itself and its treatment, also suffer adverse effects on their psychological and social functioning^26^. Vaginal aging also exacerbates of depressive symptoms, further affecting women’s emotional health and social participation abilities^27^. Therefore, treatments targeting vaginal aging represent a substantial physical and psychological improvement for women overall.

## Population Involved in Vaginal Aging

### Reproductive System–Related Factors

**Fig. 2:**
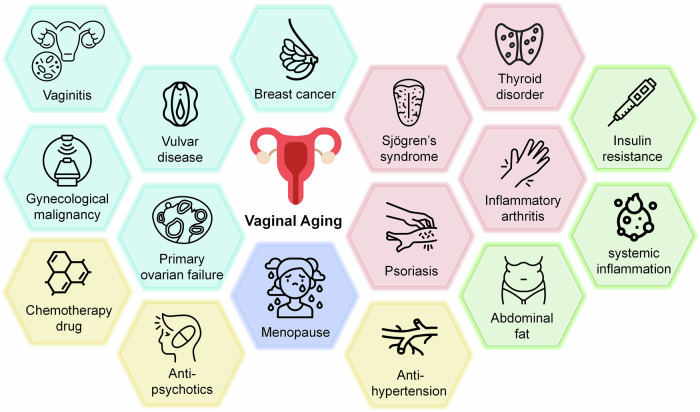
Schematic diagram of the population spectrum affected by vaginal aging. The primary population (blue) includes perimenopausal and postmenopausal women, where estrogen decline drives physiological vaginal aging. Colored hexagons classify risk factors for vaginal aging: light blue = reproductive system-related factors, pink = non-reproductive systemic disease-related factors, green = metabolic syndrome, yellow = medication-related factors.

The majority of symptoms associated with vaginal aging occur in perimenopausal and postmenopausal women due to estrogen decline (Fig. 2)^27^. Moderate-severe vaginal aging symptoms are common in 42% of premenopausal women^28^. With recent changes in the disease spectrum and advancements in therapeutic approaches, vaginal aging has also been observed in women of other age groups or pathological conditions.

Women with primary ovarian failure (POF) experienced poorer lubrication and increased dyspareunia compared with those with normal gonadal function^29^. These differences persisted even among women who received systemic estrogen therapy. For women with lichen sclerosus of the vulva, vaginal aging symptoms constitute a common reason for seeking medical care^30^. Patients with vaginitis exhibit accelerated vaginal aging, which may be attributed to significant differences in the diversity and composition of the vaginal microbiome compared with healthy women^31^. Patients with gynecological malignancies frequently exhibit vaginal epithelial atrophy and a significant reduction in lubricating secretions after radiotherapy^32,33^. Anti-estrogen drugs are commonly used in breast cancer treatment to block estrogen receptor signaling pathways or inhibiting estrogen synthesis. As a result, women who have survived breast cancer frequently suffer from vaginal dryness, greatly affecting their quality of life^34^. These results highlight the importance of focusing on maintaining vaginal health during cancer treatment planning.

### Non-Reproductive System Disease–Related Factors

Many non-reproductive diseases can also affect vaginal aging. For example, compared with healthy control groups, women with primary Sjögren’s syndrome undergo accelerated vaginal aging, particularly with lower scores in sexual function^35^. The same phenomena apply to thyroid autoimmune disorders^36^, inflammatory arthritis^36^, and psoriasis^37^.

Metabolic syndrome is a highly prevalent public health concern worldwide. Emerging evidence suggests that this metabolic disorder may also contribute to accelerated vaginal aging^38^. Pro-inflammatory factors secreted from adipose tissues and macrophages may act on the vaginal mucosa through blood circulation, impair its immune defense function and disrupt the dominant position of Lactobacillus^39,40^. Insulin resistance is the core pathological feature of metabolic syndrome. It may block insulin signaling pathways in vaginal epithelial cells and fibroblasts, thereby inhibiting glycogen synthesis, cell proliferation and differentiation^41^. Diabetes also significantly affects vaginal health. Although microvascular density is increased in the vaginal tissue of diabetic patients, the blood vessels show destructive morphological changes^42^. Meanwhile, the expression of androgen receptor and nitric oxide synthase in the vaginal epithelium and lamina propria is significantly decreased^42^.At present, studies on the impact of metabolic syndrome on vaginal aging remain insufficient. Nevertheless, this field represents an important research direction that urgently requires attention.

### Medication-Related Factors

Some drug treatments will also inevitably affect the vaginal function. Anti-psychotics may induce vaginal aging symptoms, like vaginal lubrication^43,44^. One of the primary pathways involves the elevation of prolactin levels, which can disrupt the normal hormonal balance necessary for sexual arousal and function^45,46^. Chemotherapy drugs also cause inflammation and fibrosis of the vaginal mucosa, which further exacerbate vaginal aging^47^. Interestingly, the diversity of the vaginal microbiome significantly increases in cervical cancer patients undergoing chemotherapy, which may be related to the antibacterial effects^48^. Traditional anti-hypertensive medications, such as diuretics and β-receptor blockers are known to negatively impact sexual function^49^. While most of this research focus on male populations, limited studies involving women indicate these medications could negatively impact vaginal aging^49^.

As a whole, vaginal aging affects increasing populations and exhibits significant impact on quality of life. It suggests an urgent need to re-emphasize the clinical and public health importance of vaginal aging. As such, biomarkers are not merely useful tools but indispensable enablers for transitioning vaginal aging from a underrecognized topic to a biologically defined, precisely treatable one.

### Biomarkers of Vaginal Aging

**Fig. 3:**
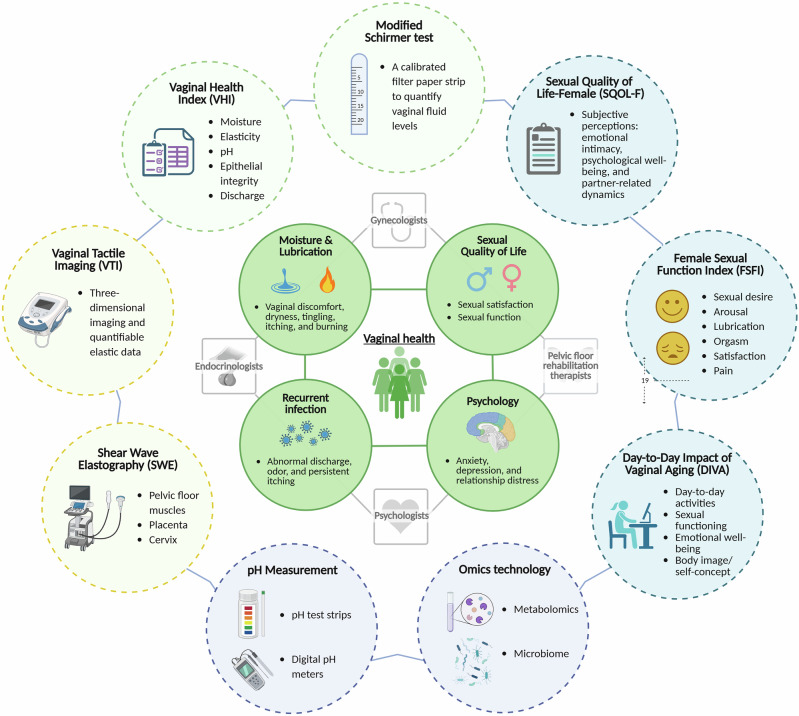
Multidimensional assessment tools for physiological characteristics of vaginal aging.

Vaginal aging is characterized by distinct clinical symptoms and measurable biomarkers, with the former representing subjective or observable phenotypic manifestations and the latter referring to quantifiable biological parameters. In the following sections, we first clarify the core clinical symptoms of vaginal aging, and then classify the vaginal biomarkers into four categories: biochemical, imaging, histological, and molecular biomarkers (Fig. 3). As a whole, clinical symptoms are the overt manifestations of vaginal aging and provide a clinical basis for the detection and validation of biomarkers.

## Clinical Symptoms

### Vaginal Dryness and Impaired Lubrication

**Fig. 4:**
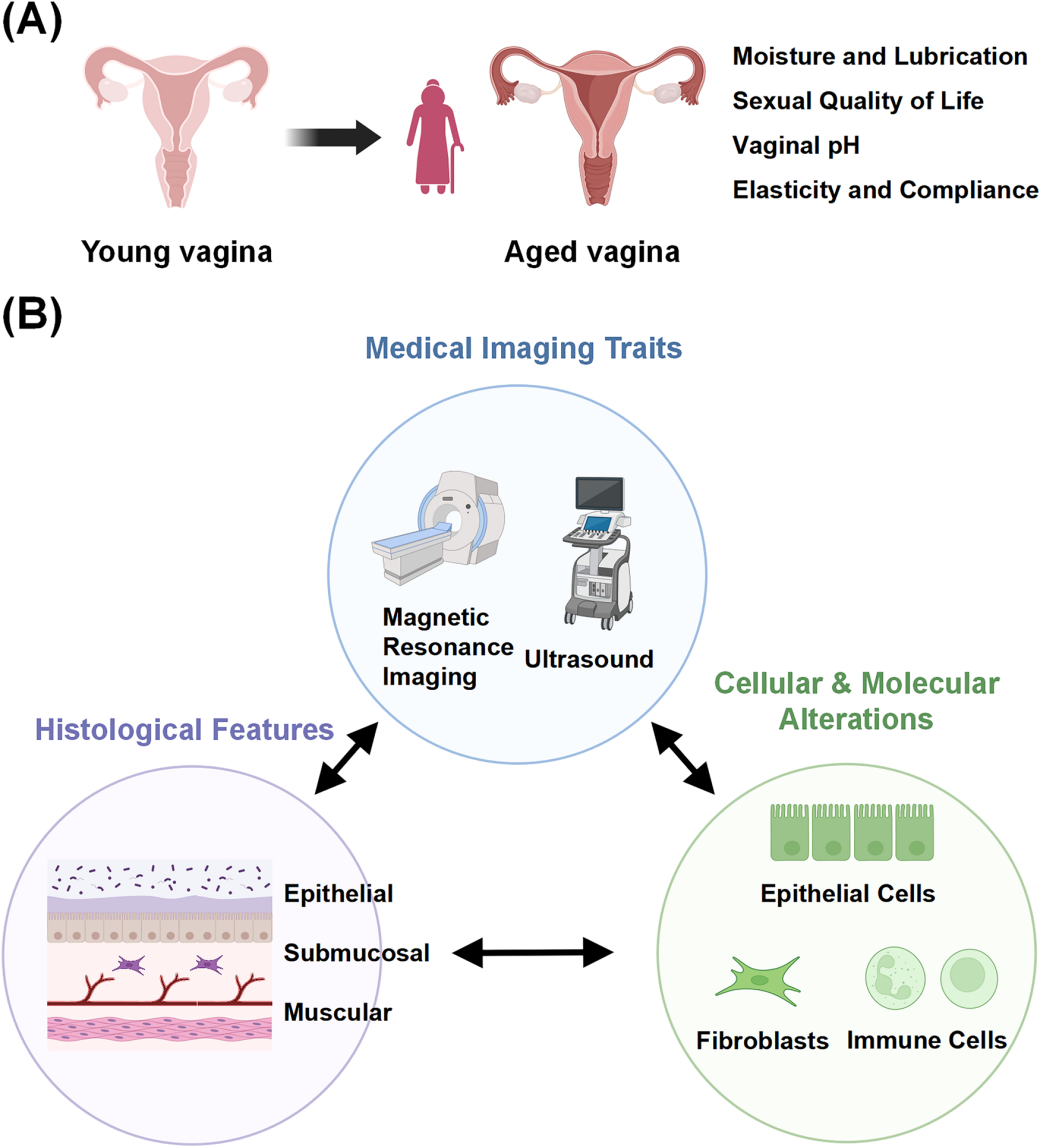
Representative biomarkers of vaginal aging indicating physiological symptoms and structural changes. (**A**) Physiological symptoms of vaginal aging. (**B**) Medical imaging traits, histological features and cellular alterations of vaginal aging.

Unlike vasomotor menopausal symptoms, vaginal aging progresses gradually and rarely improves without therapeutic intervention (Fig. 4A)^50^. According to the International Vaginal Health: Insights, Views and Attitudes study, 83% of individuals experiencing vaginal discomfort report dryness, with tingling affecting 27%, itching affecting 26%, and burning affecting 14%^23,51^. The true prevalence might be higher since most women avoid disclosing vaginal symptoms. Vaginal aging is primarily caused by the decline in estrogen levels, which reduces the number and function of vaginal epithelial cells, leading to decreased secretion of mucus and glycogen^52^. The reduced vaginal moisture not only causes discomfort (e.g., itching, burning, and irritation) but also impairs sexual function by reducing vaginal lubrication during sexual arousal^53^.

Clinical assessment of vaginal moisture can be performed using either subjective questionnaires or objective measurements. The Vaginal Health Index is a widely used tool that evaluates five parameters: vaginal moisture, elasticity, pH, epithelial integrity, and discharge, with scores ranging from 0 (severely) to 20 (normal)^54^. Epithelial integrity reflects the vaginal mucosal barrier function against pathogens^55^, and discharge status helps identify potential infections or pathological conditions^56^.

One promising objective method for assessing vaginal moisture is the modified Schirmer test, which uses a calibrated filter paper strip to quantify vaginal fluid volume. This method is adapted from the ophthalmic Schirmer test originally designed for measuring ocular moisture. It demonstrated a significant difference in moisture levels between GSM women with or without vaginal dryness. Furthermore, strong correlations were found between the test results with vaginal pH and the Vaginal Health Index, supporting its utility as a reliable objective measure^57^. The modified Schirmer test revealed significantly lower moisture levels in mice with reduced estrogen, paralleling the conditions observed in postmenopausal women^58^. Additionally, the modified Schirmer test is sensitive enough to detect increases in physiological lubrication in response to sexual stimuli^59^. This further emphasizes the versatility of the modified Schirmer test as a tool for evaluating various aspects of vaginal health. The combination of subjective and objective assessments allows for a more nuanced understanding of vaginal health, facilitating personalized treatment approaches.

### Dyspareunia

Vaginal aging has negative impacts of on sexual quality of life, as can be observed in postmenopausal women^60^. A study on gynecological cancer survivors with vaginal aging found that 67% of sexually active women reported dyspareunia, with 55% experiencing superficial pain, 40% deep pain, and 36% both^33^. Vaginal aging exerts an adverse impact on sexual arousal, libido, orgasm, and satisfaction in sexual life, although these can be improved via vaginal estrogen therapy^61^.

Sexuality is a deeply personal experience, shaped by an individual’s life history, relationship dynamics, and physical and psychological well-being^62^. Many studies have emphasized the intimate interplay of personal and relational factors in shaping sexual experiences^63–65^. Therefore, sexuality is not merely a biological drive but is deeply embedded in the emotional and relational fabric of individuals’ lives. Assessing sexual quality of life involves multiple dimensions and sensitivity. Interpretation of the assessment findings should consider the individual’s environment, relationship patterns, social support, cultural norms, and personal values.

The Sexual Quality of Life-Female captures women’s personal perceptions of their sexuality, including emotional connections, psychological well-being, and dynamics with partners,^66^. It highlights the psychosocial aspects and personal fulfillment associated with sexual life, offering insight into their overall sexual quality of life. The Female Sexual Function Index covers aspects such as sexual desire, arousal, lubrication, orgasm, satisfaction, and pain, providing a thorough evaluation of sexual function^67^. This instrument has demonstrated its utility across different populations in both clinical and research contexts^68,69^. It has also been adapted and validated in multiple cultural contexts, such as the French^70^ and Romanian versions^71^, further supporting its global applicability.

The Day-to-Day Impact of Vaginal Aging (DIVA) survey measures the specific effects of vaginal symptoms due to menopause over the last month^72^. This instrument is structured around four subdomains: day-to-day activities, sexual functioning, emotional well-being, and body image/self-concept, each evaluated using a 5-point Likert scale. It is one of the patient-reported outcome measures advised for postmenopausal women with an evidence level of A^73^. The DIVA emotional well-being scale shows a moderately strong correlation with depression scores, while the DIVA sexual functioning scale has a strong correlation with sexual function and distress measures^27^. Studies on the Turkish and German version of the DIVA further provide compelling evidence for the DIVA questionnaire’s convergent and divergent^16,74^, demonstrating its effectiveness in assessing the impact of vaginal aging on various aspects of women’s lives.

## Biochemical Biomarkers

### Vaginal pH

The vaginal pH is a key indicator of vaginal health, reflecting the balance of the vaginal microflora. Before menopause, the vaginal pH is typically acidic (3.8-4.5), maintained by the fermentation of glycogen in vaginal epithelial cells by lactobacilli^75,76^.

Vaginal pH can be measured using pH test strips or a digital pH meter. The pH test strip is a simple and cost-effective method that allows for quick assessments. These strips are particularly useful in detecting bacterial vaginosis^77,78^. However, lighting conditions can affect the accuracy of pH test strip results. Recently, machine learning has been applied to pH test strip analysis by extracting artificial features from strip images, enabling highly reliable quantitative pH prediction^79^. Another study introduced a smartphone-based system, known as the Smart-pH-Reader. By identifying the dominant or complementary wavelength of the color image and utilizing a 3D-printed optical accessory for image quality assurance, this technique achieved a quantitation precision of 0.05 pH units^80^. Overall, incorporating machine learning methods into the quantitative assessment of pH values opens up more dependable and accessible pH measurement options.

Digital pH meters have been used to investigate the association between vaginal pH and reproductive health outcomes, including misoprostol efficacy in midtrimester abortion and susceptibility to HPV infection^81,82^. Notably, vaginal pH can be affected by menstruation, sexual intercourse, and the use of vaginal products, so measurements should be performed under standardized conditions (e.g., 3-5 days after menstruation, without sexual intercourse or vaginal product use for 24 hours before measurement)^83,84^.

### Metabolites

Vaginal secretions and cervicovaginal lavage fluid are the main non-invasive sample sources for metabolomic analysis. The study of atrophic vaginitis through untargeted metabolomics has revealed significant alterations in the metabolic profiles of affected individuals compared to healthy controls. This research identified 561 differential metabolites, primarily concentrated in fatty acids, glycerophospholipids, and steroids, which are enriched in pathways such as purine metabolism and choline metabolism^85^. By combining microbiome, immunoproteome, and metabolome data, sphingolipids and long-chain unsaturated fatty acids were identified as strong predictors of genital inflammation, while amino acid metabolism was more closely associated with alterations in vaginal microbiota and pH^86^. A significant reduction in glycogen and lactic acid levels with aging were observed as well^87^. Lipoxygenase enzymes become more active with advancing age and may increase the levels of lipid peroxides^88^. In vaginal tissues, elevated lipid peroxides could exacerbate the degradation of cellular components, leading to the thinning and atrophy often observed in postmenopausal women.

### Imaging Biomarkers

Medical imaging techniques provide a non-invasive way to visualize the structural changes of the vagina with aging, complementing clinical examination and histological analysis (Fig. 4B).

### Traditional Ultrasound

Ultrasound is a widely used imaging technique in gynecology for its portability, low cost, and free of ionizing radiation. The above mentioned VTI and SWE are both innovative ultrasound-based techniques. The standardized three-dimensional ultrasound method for measuring vaginal wall thickness enables precise measurements across different anatomical planes with high repeatability and reliability^89^, as well as the anterior and posterior vaginal wall^90^. When evaluating vaginal laxity, abdominal and transvaginal ultrasound approaches observed significant differences in wall thickness measurements at various anatomical sites^91^. Transabdominal ultrasound measurements revealed that postmenopausal women exhibited significantly thinner vaginal walls compared to their premenopausal counterparts^92,93^, and it show a strong correlation with the Vaginal Health Index^94^.

### Elasticity Imaging Technology

Vaginal elasticity and compliance are important for sexual intercourse and childbirth, as they allow the vagina to stretch and return to its original shape^33^. With aging, the collagen and elastin fibers decrease in vaginal wall, leading to reduced elasticity and compliance. Clinical assessment of vaginal elasticity and compliance is often subjective, based on the gynecologist’s digital examination. Advanced imaging methods, including vaginal tactile imaging (VTI) and shear wave elastography (SWE), play a key role in evaluating tissue elasticity. VTI functions like manual palpation but improves it by offering 3D imaging and measurable data on tissue elasticity^95^. VTI finds that vaginal tissue elasticity is significantly decreased in pelvic organ prolapse, with the anterior and posterior compartments exhibiting lower elasticity in Stage III prolapse than in normal conditions^95^. On the other hand, SWE showed that women in the vaginal aging group had a significantly lower vaginal elasticity index compared to those in the non-vaginal aging group^96^. Indeed, it is frequently employed to measure the elasticity of pelvic floor tissues, providing valuable information on postpartum recovery^97^. Recently, SWE has even been employed to evaluate placental elasticity in pregnancies affected by Trisomy 21^98^. It also found that cervical elasticity changes significantly across trimesters^99^, and correlates cervical elasticity with menstrual pain^100^.

### Magnetic Resonance Imaging (MRI)

MRI facilitates clear visualization of the anatomical structure of pelvic organs and the pathological lesions. Theoretically, MRI could be used to assess changes in vaginal wall thickness, tissue architecture, and surrounding tissues, thereby providing imaging evidence for the evaluation of vaginal aging. However, to date, there have been no clinical studies specifically designed to systematically and comprehensively investigate vaginal aging using MRI.

### Histological Biomarkers

The vaginal wall consists of three layers: the mucosal layer, the muscular layer, and the adventitial layer^101,102^. Histological analysis of vaginal tissue provides detailed information about the structural changes of the vagina with aging.

### Epithelial Layer Changes

The innermost layer of the vaginal wall is a non-keratinized stratified squamous epithelium supported by a highly vascular network within connective tissue. During vaginal aging, the thickness of the vaginal epithelium is significantly reduced, and the maturity of epithelial cells is decreased—characterized by an increase in basal cells and a decrease in superficial cells^103^. This is primarily attributed to the estrogen deficiency, leading to abnormal proliferation of epithelial cells and failure of keratinized differentiation via mTORC1 signaling pathway^104^. Concurrently, the expression of estrogen receptor α in vaginal epithelial cells is also downregulated, which impairs the cellular responsiveness to estrogen. This, in turn, disrupts the normal metabolism and function of the vaginal epithelium^103^. Epithelial changes compromise vaginal lubrication, elasticity, and barrier function, adversely affecting women’s reproductive health and increasing susceptibility to pathogen invasion.

### Submucosal Layer Alterations

The aging of the vaginal mucosa affects multiple tissues and cellular structures and functions. Blood vessels in the submucosal layer become atrophy, leading to reduced blood supply, which in turn diminishes nutrient delivery and metabolism in vaginal tissues^42^. A notable shift occurs in the collagen subtype ratio, specifically an increase in the Type I/III collagen ratio, indicative of a fibrotic process^105^. A study using scanning electron microscopy revealed that vaginal aging accompanies a less organized and more fused network of collagen fibrils^106^. Similarly, increased elastin and collagen density are found in radiotherapy-induced pathological vaginal aging^107^. The immune landscape of the vaginal submucosal layer also changes with age. Research has shown that there is a significant decrease in the total number and percentage of CD4 + T cells in the aged vagina, especially post-menopause. This decline is accompanied by an increase in Th1 cells and a decrease in Th17 and Treg cells, which are crucial for maintaining mucosal immunity^108^. These changes collectively reflect the multiple degenerative changes in blood supply, structural support, and immune protection during vaginal aging, and also provide important references for intervention research.

### Muscular Layer Changes

This layer is primarily composed of smooth muscle fibers, which are essential for the support and function of the pelvic floor. With aging, vaginal smooth muscle cells undergo a reduced number and diminished function. These changes lead to muscle fiber atrophy, as well as decreased vaginal contractile function and increased vaginal stiffness^109^. It is intriguing that knocking out LOXL1 leads to greater stiffness in pre-confluent cultures and reduced stiffness in confluent cultures of vaginal smooth muscle cells. It implies that the mechanical integrity of the vaginal connective tissue is affected by ECM crosslinking^110^. Therefore, the muscular layer of the vagina is a dynamic structure influenced by various factors, including ECM composition, hormonal changes, and anatomical configuration.

### Molecular Biomarkers

Molecular alterations are the underlying cause of the structural and functional changes of vagina aging. These alterations include changes in the number, function, and phenotype of vaginal epithelial cells, fibroblasts, and immune cells.

### Epithelial Cells

Vaginal epithelial cells are the primary cells of the vaginal epithelium, responsible for producing glycogen, maintaining the acidic vaginal environment, and providing a physical barrier against pathogens. During vaginal aging, the proliferative capacity of vaginal epithelial cells reduced and the expression of cell cycle-related proteins are altered, leading to thinning of the epithelial layer^103^. Experimental data showed that the expression of aquaporins in vaginal epithelial cells was downregulated, accompanied by a reduction in vaginal lubrication^111^. However, the function of vaginal epithelial cells can be restored via the VDR/p-RhoA/p-Ezrin signaling pathway after vitamin D supplement^112,113^. Vaginal epithelial cells also undergo metabolic changes with aging. Glycogen production by vaginal epithelial cells is significantly reduced with age, especially after menopause^114^. The glycolytic pathway and oxidative stress both increased, and they are closely associated with decreased intracellular mitochondrial function^115,116^. These metabolic alterations may further accelerate the aging of vaginal tissues by influencing cellular proliferation and apoptosis pathways^117^. Notably, age-related changes in epithelial cell composition are also critical. Aged vaginas show an increased proportions of basal epithelial cells and decreased intermediate epithelial cells, reflecting reduced epithelial maturity^118^.

### Fibroblasts

Fibroblasts undergo significant changes during vaginal aging. When vaginal fibroblasts from elderly women were reprogrammed into induced pluripotent stem cells, their expression of aging markers showed no significant difference compared to younger women, indicating limited age-related impact on these cells’ reprogramming capacity^119^. In postmenopausal women, the expression of all matrix metalloproteinases shows a downregulated trend, whereas the expression of tissue inhibitors of metalloproteinases and lysyl oxidase families is significantly upregulated^120^. The oxidative stress induces collagen degradation and cellular senescence in vaginal fibroblasts, which can be mitigated by autophagy mechanisms^121^. Furthermore, the stiffness and composition of the extracellular matrix impede vaginal fibroblast differentiate into myofibroblasts, forming a vicious cycle in vaginal tissues^122^. An interesting discovery is the presence of “inflamed fibroblast” subpopulations in aged vaginas. These fibroblasts show elevated activity of inflammation-associated transcription factors (e.g., NFKB1, RELA) and ECM regulatory transcription factors (e.g., Wt1, Klf5), creating a self-sustaining pro-fibrotic microenvironment^118^.

### Immune Cells

Immune cells play a crucial role in maintaining vaginal health by defending against pathogens and regulating inflammation. Fluctuations in estrogen and progesterone regulate vaginal neutrophil migration and activity during vaginal aging, thereby modulating the reproductive tract’s immune response^123,124^. The density of Langerhans cells may also reduce, and disrupt the antigen presentation and immune activation function, weakening the vaginal defense ability^125^. Meanwhile, the increasing number of activated T cells in the aging vagina indicates that the body is transitioning toward a state more prone to inflammation^126^. The cytokines secreted by immune cells also undergo changes, leading to an imbalance in inflammatory responses. FOLR2⁺ macrophages, a subtype enriched in aged vaginal tissues (>60 years), highly express pro-inflammatory factors such as TNF-α, IL-6, and CXCL1^127^. HIV-negative postmenopausal women showed markedly lower levels of MIP-3α, IL-6, and SLPI than premenopausal women^128^. Pro-inflammatory cytokines like IL-1β, IL-6, and IL-8, when present in higher concentrations, can suppress the growth of beneficial Lactobacillus species and promote the proliferation of opportunistic pathogens^129^.

### Microbiome Alterations

The vaginal microbiome is a key regulator of vaginal microenvironment homeostasis. Lactobacillus is the dominant genus in the healthy premenopausal vaginal microbiome, while vaginal aging is characterized by a loss of Lactobacillus crispatus and Lactobacillus iners^130^. Concurrently, an increase in anaerobic bacteria such as Gardnerella, Prevotella, and Atopobium, accompanied by increased microbial diversity and the formation of polymicrobial biofilms on the vaginal epithelium were demonstrated during vaginal aging^130,131^. Notably, the postmenopausal women exhibit the highest evenness of vaginal microbiota and a shift toward an Ehrlich “Rivet” model, where species are functionally interrelated and evenly distributed^132^.

## Challenges and Future Perspectives

Despite the significant progress in the identification and application of vaginal aging biomarkers, several challenges remain.

### Lack of Standardization

There is a lack of standardization in the measurement of vaginal aging biomarkers, including differences in sample collection methods (e.g., vaginal secretions, tissue biopsies), measurement techniques (e.g., pH test strips, digital pH meters), and reference ranges. This issue is a common theme across various domains of aging research. For instance, different sample collection methods, such as venous blood, finger prick dried blood spots, and saliva, implies inconsistency in using telomere length as a biomarker of cellular aging^133^. Alcazar et al. proposes a standardized six-step method to measure vaginal wall thickness: filling the vagina with gel to create an acoustic window, inserting the endovaginal transducer to the vagina’s middle third, capturing a 3D volume of the vagina’s proximal third in the sagittal plane, obtaining at least three 1 cm-separated axial planes, magnifying the image by 1.5, and measuring thickness at 12, 3, 6, 9 o’clock in the second axial plane. The study highlights the importance of consistent methodologies in obtaining reliable data^89^. The method includes six steps: To sum up, the standardization of aging biomarkers is essential for enhancing their reliability and comparability across studies and clinical practices^134,135^. Efforts to harmonize biomarker measurements, establish reference systems, and develop standardized validation protocols are critical for advancing the field of aging research and improving the clinical utility of biomarkers.

### Limited Understanding of Mechanisms

Contemporary omics technologies have become powerful tools for exploring the molecular mechanisms of aging and screening specific biomarkers, but their application in vaginal aging is still limited. There is a lack of transcriptomic and proteomic research on human vaginal aging tissue, and the results cannot be directly translated to human clinical practice. The exact mechanisms that cause vaginal aging are not entirely clear, which limits the identification of specific and sensitive biomarkers. For instance, in the context of neurodegenerative diseases, ultrasensitive detection techniques have been pivotal in elucidating the mechanistic pathways involved, thereby facilitating the identification of relevant biomarkers^136^. Similarly, the exploration of mitochondrial and ferroptotic mechanisms in systemic lupus erythematosus helps to develop predictive diagnostic models with high accuracy^137^. As research continues to evolve, the integration of mechanistic insights will remain a cornerstone in advancing personalized medicine and the improvement of patient outcomes.

### Exploration of Systemic Links

Future research should explore the links between vaginal aging and systemic aging, such as the relationship between vaginal atrophy and cardiovascular disease, osteoporosis, and cognitive decline. This challenge is rooted in the complex interplay between localized aging processes and systemic physiological changes. The vaginal environment undergoes specific changes due to hormonal fluctuations, particularly during menopause, which can lead to increased inflammation and alterations in the microbiome^126^. These changes are not isolated but are part of a broader systemic aging process that affects multiple organ systems. Exploration of systemic links will lead to the development of biomarkers that reflect not only local vaginal changes but also systemic effects of vaginal aging, providing a more comprehensive assessment of women’s health.

### Vaginal Microbiome as a Promising Biomarker

The vaginal microbiome is one of the most promising biomarker domains for vaginal aging. It integrates multiple layers of biological information—including hormonal status, epithelial barrier function, and immune regulation—making it a comprehensive and sensitive indicator of vaginal aging. Microbiome profiling via vaginal swabs is easily implementable in routine clinical practice. The modifiability of the microbiome via probiotic supplementation, lactobacillus transplantation, or targeted antimicrobial therapy renders it a potential therapeutic target. Restoring Lactobacillus dominance may not only correct microbiome imbalance but also reverse or mitigate downstream vaginal tissue degeneration. For example, supplementation with Lactobacillus crispatus has been shown to enhance vaginal acidification and reduce pathogenic bacterial colonization, and improve epithelial barrier function in postmenopausal women^138,139^. In a randomized controlled trial, the administration of L. crispatus significantly reduced the recurrence of BV after antibiotic treatment^140^.

## Conclusion

As a pivotal component of the female reproductive system, the vagina undergoes distinct aging-related changes that are closely intertwined with women’s physical health, sexual well-being, and psychological quality of life. Vaginal aging biomarkers hold substantial promise for transforming clinical practice by enabling early identification of subclinical changes and personalized intervention. This review provides a comprehensive overview of currently available evidence regarding biomarkers associated with vaginal aging, thereby filling critical gaps in the field of women’s health and aging research. It also serves as a valuable reference for researchers, clinicians, and public health professionals, with the aim of accelerating biomarker development and improving health outcomes for women affected by vaginal aging.

## Data Availability

No additional data are available.
